# Accuracy of Implant Guided Surgery in Fully Edentulous Patients: Prediction vs. Actual Outcome—Systematic Review

**DOI:** 10.3390/jcm13175178

**Published:** 2024-08-31

**Authors:** Mafalda Azevedo, Francisco Correia, Ricardo Faria Almeida

**Affiliations:** 1 Faculty of Dental Medicine, University of Porto, 4200-393 Porto, Portugal; m2.azevedo2024@gmail.com; 2 Specialization in Periodontology and Implants, Faculty of Dental Medicine, University of Porto, 4200-393 Porto, Portugal; franciscodcorreia@gmail.com; 3 Associated Laboratory for Green Chemistry (LAQV) of the Network of Chemistry and Technology (REQUIMTE), 4050-342 Porto, Portugal

**Keywords:** dental implant, image-guided surgery, computer-assisted surgery, accuracy

## Abstract

**Objectives:** Examine deviations between the digitally planned and actual implant positions in clinical studies using static fully guided surgical guides. Identify potential associated factors and strategies to minimize their likelihood. **Materials and Methods:** This systematic review was conducted following the PRISMA checklist. The literature search was conducted in the PubMed^®^ and Scopus^®^ databases up to February 2024 following the PICOS search strategy. Clinical trials conducted between 2013 and 2024, evaluating the accuracy of static fully guided surgical guides placed in fully edentulous patients, were included. The studies had to assess at least two of the following parameters: angular deviation, cervical deviation, apical deviation, and depth deviation. **Results**: Out of the 298 articles initially searched, six randomized clinical trials and three clinical trials were included. All but one article used mucosa-supported guides; the remaining one used bone-supported guides. Apical deviations were more significant than cervical deviations, and implants tended to be placed too superficially. The greatest mean deviations were 2.01 ± 0.77 mm for cervical and 2.41 ± 1.45 mm for apical deviations, with the largest angular deviation recorded at 4.98 ± 2.16°. **Conclusions:** The accuracy of the surgical guide is influenced by various factors, including the technique of image acquisition and subsequent planning, guide support methods, and the adopted surgical protocol. Apical deviations are influenced by cervical and angular deviations. Additionally, deviations were more pronounced in the mandible. Further studies with similar methodologies are necessary for a more precise assessment of the different factors and for establishing safety margins.

## 1. Introduction

Healthcare is an ever-evolving field, with significant progress made in imaging diagnostics, particularly with the integration of cone beam computed tomography (CBCT). This technology, used in various dentistry fields, offers three-dimensional imaging of a patient’s oral structures, facilitating a high-quality and precise preoperative diagnosis, especially useful for implant placement, while also minimizing the patient’s radiation exposure [1,2,3].

The digital images obtained combined with computer-aided design software (CAD) can be transformed into a virtual 3D model of the patient’s oral cavity. This model can then be used in computer-aided manufacturing (CAM) to create dental prosthetics, including immediate dentures. In order to generate the virtual model of the patient, it is necessary to merge the CBCT data (in DICOM format) with an intraoral surface scan or an extraoral scan of the stone cast (in STL format). The precise fusion of both data sets is crucial for accurately planning the implant position [4].

To obtain the CBCT data in edentulous cases to plan implant position, two methods are described [5]. In the single scan method, a duplicate of the future prosthesis, designed to closely resemble the final prosthesis, is created. This replica, which contains radiopaque material, is then used during the CBCT scan. Alternatively, in the double scan method, radio-opaque markers are fixed to the scan prosthesis. Initially, the prosthesis is scanned alone, followed by scanning the patient wearing the prosthesis. The markers are securely attached as the prosthesis is inserted into the mouth using a rigid bite index. Subsequently, the two data files are merged for planning purposes [5].

To acquire a patient’s oral scan, two methods are commonly used [6]. The first involves indirectly scanning a conventional impression, which is poured into gypsum, using a laboratory extraoral scanner (EOS). Alternatively, intraoral scanners (IOSs) are increasingly being utilized. This technique relies on the capability of scanners to capture intraoral tissues and then align these images with the CBCT scan data. Factors such as lighting conditions, the IOS’s calibration, tissue mobility, and the dentist’s skill all influence the scanning accuracy [6,7,8].

A realistic representation of the patient’s bone structure and oral cavity enables the practitioner to perform a virtual simulation of the surgery in an ideal and precisely prosthetically guided way [9]. In fact, the capability to plan inter-implant distance, implant depth, and other factors has elevated implant planning to a pivotal tool for ensuring optimal outcomes [10].

Following virtual implant planning, two distinct techniques can be utilized to transfer the planned implant positions to the surgical site, namely the static and the dynamic approach [11].

The static method involves utilizing a prefabricated surgical template, a physical tool, that is designed to direct the drills in accordance with the digitally predetermined position. This method does not permit adjustments to the implant position during surgery [5,10]. On the other hand, the dynamic approach utilizes optical motion tracking technology to enable real-time guidance during surgery, allowing the operator to alter the implant position during surgery [5,10,11].

To ensure a precise alignment of the implants according to the planned direction, the drills must fit perfectly into the guide holes of the surgical guide. In fully guided surgeries, a restrictive design is used, limiting the movement of the drills from the initial osteotomy to the final drill. On the other hand, partially guided surgical guides involve initial drilling restricted by the guide, which is then removed, allowing the rest of the procedure to be performed by the clinician without a guide. In general, fully guided surgical guides are more commonly used in fully edentulous patients [12].

There are many challenges regarding the use of a surgical guide in fully edentulous patients. Edentulous jaws present a tissue-based clinical scenario characterized by multiple mobile areas and smooth surface textures entirely coated by saliva, which complicates intraoral scanning. Additionally, the absence of reference points in edentulous jaws interferes with calibrating the patient’s data on the CAD-CAM software [13].

In fact, it is widely accepted that guided surgery in partially edentulous patients demonstrates higher accuracy compared to guided surgery in fully edentulous patients [5,10].

Two types of surgical guides can be used in fully edentulous patients, namely bone-supported and mucosa-supported surgical guides [5].

Bone-supported surgical guides are placed directly on the bone after opening a mucoperiosteal flap. They were the first type of surgical guides used in edentulous patients, and they offer the key benefit of providing a clear visualization of the surgical site and facilitating precise control over the drilling depth [5]. However, this type of guide involves quite an invasive surgery, which leads to postoperative discomfort, and the diminished blood supply hinders the healing process. In contrast, mucosa-supported surgical guides enable a flapless approach, potentially reducing both the overall duration of the surgery and postoperative discomfort [5,14,15].

The flapless approach necessitates a minimum of 4.5 mm to 5 mm of keratinized gingival tissue and an alveolar bone width of at least 4 to 4.5 mm [16]. Nevertheless, one significant disadvantage of this method is the inability to access and assess the surgical bone area, which might be a hardship, especially for inexperienced dentists. There is also a bigger risk of thermal damage to the tissue, due to reduced access for external irrigation [11,15,17].

In fully edentulous patients, planning for implant-supported rehabilitations is particularly important, as the absence of stable anatomical structures makes the rehabilitation of these clinical cases more complex and challenging. Therefore, it is essential to ensure the accuracy of the surgical guides in order to avoid important anatomical structures and prevent complications [1].

The term “accuracy” in implant surgery has been described as the difference between the virtual planned position and its actual placement in the mouth. These deviations can arise from various contributing factors, as they result from errors starting from digital capturing and radiographic acquisitions, extending to the conversion of the virtual design into a surgical guide and its subsequent incorrect placement in the patient’s mouth. The accuracy is commonly evaluated using radiographic techniques, namely a postoperative CBCT. This method involves superimposing preoperative and postoperative CBCT scans to measure any deviations between the planned and actual implant positions. Another approach involves the use of computer-aided comparison, where specialized software overlays the preoperative surgical plan with the postoperative CBCT images to precisely quantify deviations [18,19,20].

Thus, the precision of guided surgery relies on the interplay of all errors accumulated throughout the entire process [1,12].

As life expectancy increases, the chances of treating patients who are fully edentulous or have terminal dentition also rise [12].

Hence, this systematic review aims to examine clinical studies for discrepancies between digitally planned implant positions and their actual placement in the mouth, using a static fully guided surgical guide. Additionally, it seeks to identify potential contributing factors and strategies for minimizing the likelihood of such deviations.

## 2. Materials and Methods

This systematic review has been registered in PROSPERO with the following registration ID: CRD42024506436.

It was elaborated in accordance with the PRISMA checklist (Preferred Reporting Items for Systematic Reviews and Meta-Analyses guidelines) [21] conforming to the PICOS methodology.

An extensive electronic search was carried out in the MEDLINE (PubMed^®^) and Scopus^®^ databases up to 12 February 2024, and the search strategy was based on the following combinations:

PubMed^®^: image-guided surgery (MeSH terms) AND dental implant (MeSH terms) (fully edentulous OR edentulous OR edentulous jaw) AND (Computer assisted guided surgery OR computer aided design OR computer-assisted OR digital workflow OR Computerassisted surgery OR surgical template OR surgical guide OR Image based Surgery OR image guided surgery OR 3D Imaging) AND dental implant.

Scopus^®^: (fully edentulous OR edentulous OR edentulous jaw) AND (Computer assisted guided surgery OR computer aided design OR computer-assisted OR digital workflow OR Computerassisted surgery OR surgical template OR surgical guide OR Image based Surgery OR image guided surgery OR 3D Imaging) AND dental implant.

### 2.1. Inclusion and Exclusion Criteria

The studies found were screened based on predetermined inclusion and exclusion criteria.

**Figure 1:**
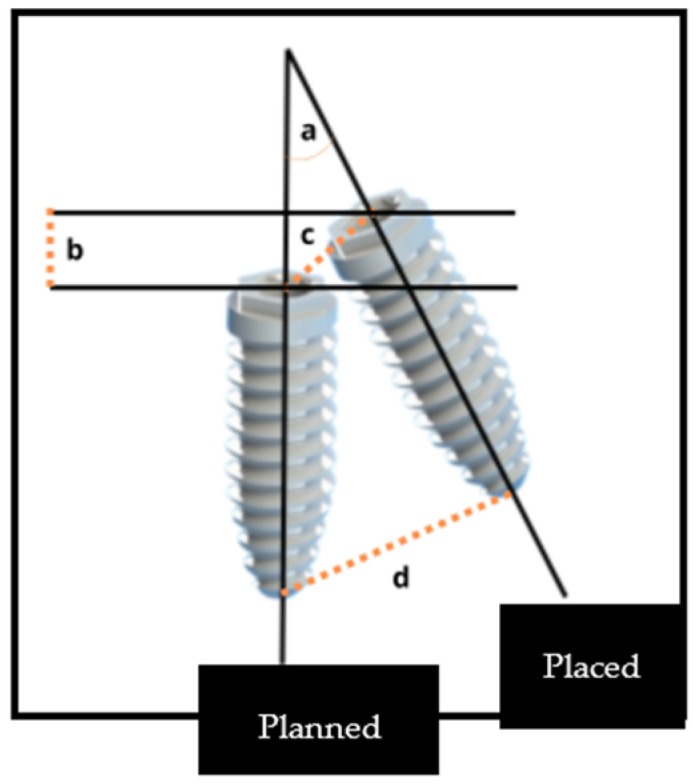
Demonstrates the characterization of the different types of deviation (image based on reference [22]). Legend: (a) angular deviation; (b) depth deviation; (c) coronal deviation; (d) apical deviation.

Inclusion criteria:

Clinical studies, clinical trials, randomized controlled trials, and controlled clinical trials with a time limit between January 2013 and February 2024, written in Portuguese, English, Spanish, or German, and studies carried out in humans were included. The surgical guides must be static, fully guided, bone or mucosa-supported, and placed in completely edentulous patients. Studies must evaluate at least two types of deviation: angular, cervical, apical, and depth deviation. Figure 1 illustrates the characterization of the various types of deviations.

Exclusion criteria:

Opinion or empirical articles, case series, cohort, or case and control studies, bibliographic or systematic reviews, meta-analyses, animal studies, articles published outside the established time limit, and unrelated articles were excluded. Furthermore, studies carried out with zygomatic, pterygoid, and orthodontic implants were excluded.

The articles were organized with the use of the reference management software EndNote 21^®^ (Clarivate Analytics, Philadelphia, PA, USA).

### 2.2. Selection of Studies

All articles were screened by two independent researchers, firstly by MA, secondly by RFA, and when in doubt, a third reviewer, FC, evaluated the articles. The articles were selected initially by title, secondly by summary, and finally after a full reading. Only studies selected by unanimous agreement among reviewers were chosen for analysis. Disagreements were solved through discussion.

**Figure 2:**
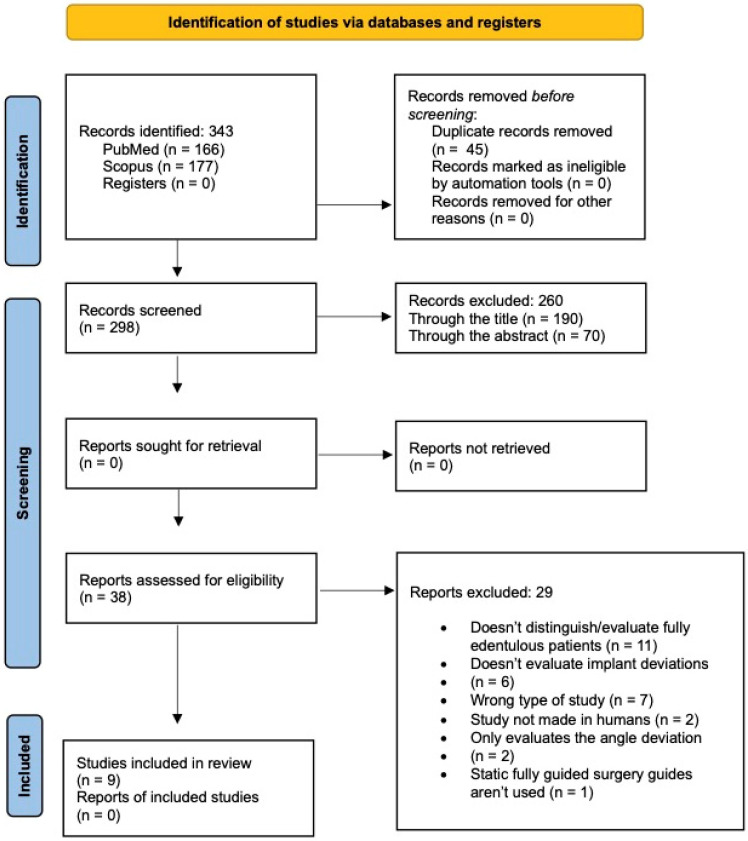
Flowchart of the electronic search and selection of studies.

The selection process is shown in the PRISMA flow diagram (Figure 2).

### 2.3. Accuracy Analysis

To evaluate the accuracy, a comparison between the planned position of the implant and its actual position post-insertion was made. Various measuring points were employed to make this comparison:

- Coronal disparity, assessed at the entry point center of the implant
- Apical disparity, assessed at the apex center
- Angular disparity
- Depth disparity

Discrepancies at the entry and apex points, as well as depth discrepancies, were all measured in mm, while angular deviations were quantified in degrees.

The predominant method of measuring the actual distance between the planned and actual points involved a 3D space. However, some studies [23,24] differentiated these measurements by also analyzing the mesiodistal and buccolingual deviations. These types of errors were considered a subgroup of the main deviation outcomes.

### 2.4. Data Extraction

A comprehensive analysis of the gathered data was made alongside the accuracy assessment. The following data were gathered from the studies:

Methods: type of study, location, number of centers, funding source, year, recruitment period.

Participants: inclusion criteria, exclusion criteria, age (mean age ± standard deviation), gender, number of patients, number of implants placed, number of implants analyzed.

Intervention: CAD-CAM software, implant system, implanted jaw, flapless surgery (yes or no), follow-up period, implant failure, transoperative complications.

Outcomes: primary outcomes (coronal deviation, apical deviation, angular deviation, and depth deviation) and secondary outcomes (mesiodistal and buccolingual deviation).

### 2.5. Risk of Bias

Two reviewers (MA and RFA) independently analyzed the risk of bias for each study.

The risk of bias for each randomized clinical trial (RCT) was assessed using the following Cochrane RoB 2 criteria [25]:

- Bias arising from the randomization process
- Bias due to deviations from intended interventions
- Bias due to missing outcome data
- Bias in measurement of the outcome
- Bias in selection of the reported result

Non-randomized clinical trials were also included in this systematic review. The ROBINS-I tool was used to assess the risk of bias in these studies by using the following criteria [25]:

- Bias due to confounding
- Bias in selection of participants in the study
- Bias in classification of interventions
- Bias due to deviations from intended interventions
- Bias due to missing data

## 3. Results

### 3.1. Study Selection

After the initial search, 343 articles were obtained, namely 166 from PubMed^®^ and 177 from the Scopus^®^ database. Since more than one search engine was used, it was necessary to eliminate duplicates, resulting in a total of 298 articles after filtering. All titles and abstracts were read, and their inclusion or exclusion was based on the pre-defined criteria previously mentioned, leading to a sum of 38 articles. The level of agreement between the reviewers was strong (k = 0.852).

The thorough reading of the remaining 38 articles resulted in the inclusion of nine articles in this systematic review, namely 6 RCTs [24,26,27,28,29,30] and three clinical trials [23,31,32] (Figure 2). The reasons for excluding the remaining 29 articles, following a meticulous examination, are detailed in the Supplementary File S1.

### 3.2. Study Characteristics

The selected studies were organized considering the following information: authors, year of publication, type of study, number of patients, mean age, CAD-CAM software, implant brand utilized and follow-up (Table 1).

In terms of contextualization, all studies were conducted at a single center. Nomiyama et al. (2023) [28], Jaemesuwan et al. (2023) [31], and Arisan et al. (2013) [30] received funding from university funds, whereas in both studies by Vercruyssen et al. (2014) (2016) [24,29], materials such as dental implants and surgical guides were provided by dental brands. Most studies had a recruitment period spanning three years.

The studies included in this systematic review had specific criteria for admitting patients. Moreover, as mentioned before, some studies not only determined 3D deviations, but also MD and BL deviations, as shown in Table 2.

### 3.3. Study Outcomes

Table 3 describes the surgical techniques used for implant placement, as well as the types of surgical guide utilized. Additionally, it provides information on the different outcomes obtained.

The majority of primary outcomes were presented as mean deviations (±standard deviation), with the exception of one study by Vercruyssen et al. (2016) [24], in which coronal, angular, and apical deviations were described using the mean and its corresponding range. Secondary outcomes were described as the median and interquartile range. Similarly, one study [26] opted to utilize the median and interquartile range to describe all of its results.

In terms of primary outcomes, it is noticeable that apical deviations exceed cervical deviations. Additionally, more than half of the studies that assessed depth deviations concurred that implants are often placed too superficially [23,24,27].

Secondary outcomes were only described in two of the nine selected studies [23,24], and those studies described the secondary outcomes in different ways.

Verhamme et al. (2015) [23] detailed the mesiodistal and buccolingual errors at various implant points, mirroring the approach used for the primary outcomes. On the other hand, Vercruyssen et al. (2016) [24] focused on the central point of the cervical portion of the implant for analyzing secondary outcomes and decided to present it as the median and interquartile range.

In all of the studies in which various fully guided static groups were analyzed, all outcomes are presented in Table 3 [26,27,29,30,32]. Nevertheless, in each case, no statistically significant differences were observed between the different groups.

In three studies [28,29,30], not all implants placed were analyzed.

In the Vercuyssen et al.’s study (2014) [29], one patient experienced restricted mouth opening and two implants could not be placed, while another patient received a smaller implant than initially planned. In the study by Arisan et al. (2013) [30], four implants could not be analyzed due to heavy beam-scattering associated with the titanium present in the implants. Lastly, Nomiyama et al. (2023) [28] did not provide a reason for not analyzing one of the implants.

All of the surgical guides were mucosa-supported with some type of fixation or stabilization pin/screw. Only Vercruyssen et al. (2014) [29] utilized bone-supported surgical guides in two groups, but as previously mentioned, the difference between mucosa-supported and bone-supported groups was statistically not significant and clinically not relevant.

Only three studies [24,28,30] reported the loss of implants.

Arisan et al. (2013) [30] documented the loss of two implants attributed to postoperative infection and mobility upon completion of the healing phase. Vercruyssen et al. (2016) [24] noted that in the delayed loading group, one implant failed to integrate. Lastly, Nomiyama et al. (2023) [28] did not provide an explanation for the loss of one implant.

### 3.4. Risk of Bias within Studies

Two different tools were utilized to determine the Risk of Bias, namely RoB 2 for randomized clinical trials and ROBINS-I for non-randomized clinical trials [25].

**Figure 3:**
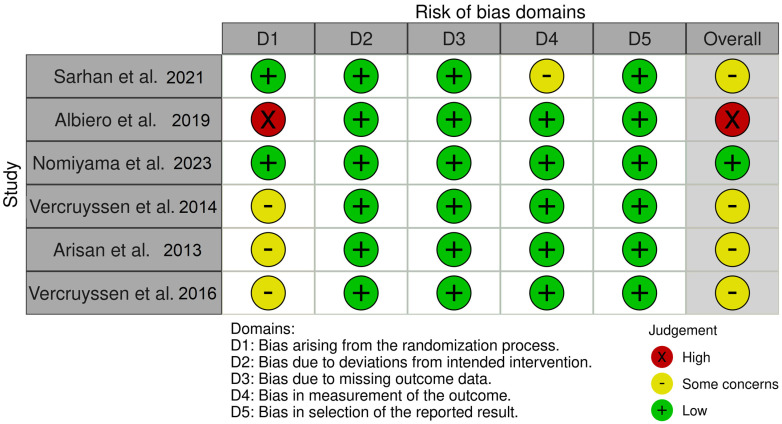
Distribution of the Risk of Bias according to the RoB 2 tool for each domain in the randomized clinical trials [24,26,27,28,29,30].

In the randomized clinical trials [24,26,27,28,29,30], most were categorized as “some concerns” since the authors inadequately reported whether the allocation sequence was sufficiently concealed (Figure 3).

One study [27] was judged to be at high risk due to the lack of a thorough randomization procedure. In the study by Sarhan et al. (2021) [26] there were some concerns about the blinding of the outcome assessors (Figure 3).

**Figure 4:**
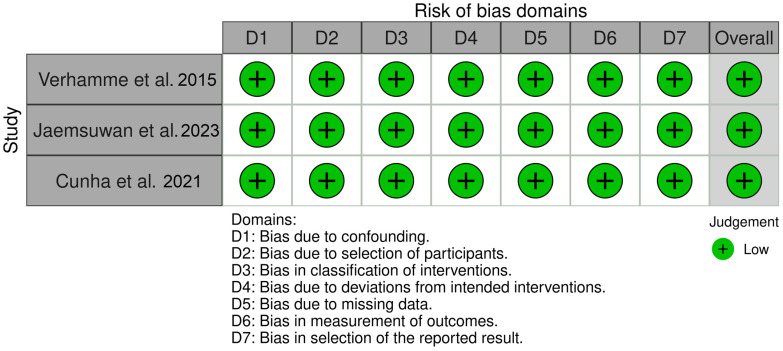
Distribution of the Risk of Bias according to the ROBINS-I tool for each domain in the clinical trials [23,31,32].

In the non-randomized clinical trials [23,31,32], all of the studies were considered to have a low risk of bias across all domains (Figure 4).

## 4. Discussion

In this systematic review, the accuracy of static fully guided implant surgery in fully edentulous patients and its associated clinical variables were analyzed.

In vitro as well as ex vivo studies were excluded from this review because they do not account for clinical factors such as limited mouth opening, patient movement, saliva, bleeding, mucosal resilience, and bone density. The inclusion of such studies might lead to an underestimation of implant position error and an overestimation of guided surgery accuracy [3,33].

In fact, Renaud Noharet et al. (2014) [34] revealed that formalin-based cadavers may undergo bone softening as a result of demineralization induced by the formalin. Additionally, it was observed that freezing cadavers might alter the properties of the mucosa. These factors can have a substantial impact on the precision of implant placement. Therefore, it would not be rational to compare implant position deviations across in vivo, ex vivo, and in vitro studies.

To comprehend the reasons behind positional deviations in implant placement using a static fully guided surgical approach, clinicians need to acknowledge and comprehend the limitations present at each stage of the digital workflow.

The first moment that can influence a possible deviation in the implant position occurs during the acquisition of the DICOM files, namely during CBCT or MSCT scans. CBCT has emerged as a popular choice in implant dentistry due to its reduced radiation exposure, affordability, and faster scanning process. However, image artifacts are more prevalent when metallic objects are present, due to the reduced dynamic range of the radiographic grayscale [1,2,3].

Arisan et al. (2013) [30] compared the deviations between intended implant positions and the actual placements guided by mucosa-supported surgical guides derived from MSCT or CBCT scans. According to the study [30], CBCT images often exhibited higher levels of noise and variability in intensity, necessitating manual adjustments of gray intensity thresholds and removal of scatter noise. Additionally, implants guided by CBCT showed slightly less accuracy compared to those guided by MSCT, although these differences were not statistically significant.

If the images used for creating the surgical guides are not clear, it can affect how accurately the planned virtual implants are transferred during surgery.

All of the studies [23,26,27,28,30,31,32] relied on CBCT to obtain the patient’s images, except for both studies by Vercruyssen et al. (2016) (2014) [24,29], in which the researchers opted to perform the preoperative scan with MSCT. According to Vercruyssen et al. (2014) [29], the initial protocol necessitated the measurement of Hounsfield Units, which was not feasible with CBCT during the study period. However, with advancements, this is now achievable. Vercruyssen et al. (2014) [29] suggest that if the study were to be conducted again, preoperative and postoperative CBCT scans would be employed to reduce radiation exposure.

The method of acquiring the CT data may also have differed between studies. Most studies preferred the double scan technique, incorporating radiopaque markers and a bite registration index in centric relation for patient scanning These elements are crucial for aligning both datasets during superimposition. Arisan et al. (2013) [30] opted for the single scan method, although a bite registration index was used to hold the guide in place.

In terms of implant planning, various software programs were utilized across the studies, with SimPlant^®^ being the most commonly used [24,27,29,30]. Throughout this process, several strategies can be employed to ensure optimal implant positioning. Arisan et al. (2013) [30] and Jaemmsuwan et al. (2023) [31] discussed the “backwards” technique, where implant positions are determined in relation to the prosthetic design. However, it is crucial to consider other factors, such as bone morphology and anatomical constraints. Verhamme et al. (2015) [23] emphasized the importance of having at least 2 mm of vestibular and lingual bone surrounding the implant. In their study [23], augmentation procedures before surgery were performed. With that in mind, it is essential to ensure that during planning, at least the implant’s tip remains within the original bone structure.

It is important to highlight that throughout the stages of image acquisition, processing, and manipulation, a margin of error of approximately 0.5 mm may arise [35]. Furthermore, it is noteworthy that incorrect configurations or settings within software applications have the potential to induce slight deformations in surgical guides, which can vary between 0.1 and 0.2 mm [36].

With image acquisition and implant planning completed, it is crucial to recognize that errors can still arise during the surgical procedure, particularly concerning the administration of anesthesia, the choice of flapless or conventional techniques, and guide fixation.

Achieving precise positioning of mucosa-supported surgical guides may be challenging due to injection-related swelling following infiltrative anesthesia [23,32].

Notably, Cunha et al. (2021) [32] observed that distortion resulting from the anesthetic infiltration could potentially have impacted the accuracy of implant positioning in their study. To mitigate this issue, Arisan et al. (2013) [30] adopted a method of slowly administering the anesthetic through the holes of the surgical guide. In contrast, Verhamme et al. (2015) [23] opted for general nasotracheal anesthesia without local anesthesia to minimize interference from swelling. However, it is important to note that the availability of general anesthesia may be limited by legal regulations in certain countries and potentially lead to higher costs.

According to several authors [27,30,32], utilizing the flapless technique during implant surgery results in a notable reduction in surgery duration, pain intensity, and post-surgery complications, bringing the procedure closer to the precision of the initial planning. However, it is crucial to adhere to the manufacturer’s instructions when performing flapless surgery [32].

Additionally, Arisan et al. (2013) [30] highlighted the importance of having a minimum of 5 mm attached mucosa for flapless surgery, as mobile non-attached mucosa may entangle with the drills, causing injury to the surrounding soft tissues. Albiero et al. (2019) [27] also suggest that immediate implant placement and restoration after tooth extraction tends to yield better aesthetic outcomes compared to implants placed in already healed sites, particularly when employing the flapless technique. Cunha et al. (2021) [32] found that flapless surgery resulted in greater accuracy than the conventional technique.

Only two studies [29,31] opted for the conventional technique, with Vercruyssen et al. (2014) [29] being the sole study to use bone-supported guides, which require a full-thickness mucoperiosteal flap.

In terms of guide stabilization, all studies employed fixation pins to secure the surgical guides in place. While the precise number of fixation screws varied among studies, most commonly utilized three or four anchor screws. Ensuring an even distribution of screws throughout the jaw, as emphasized by Vercruyssen et al. (2014) [29], is crucial for enhancing guide stability. Arisan et al. (2013) [30] went a step further, noting that osteosynthesis screws should be inserted through the designated guide openings while in full occlusion with the opposing jaw. In another clinical study conducted by Arisan et al. (2010) [37] and involving the use of multiple guides with increasing diameters, consecutive bone-supported guides frequently moved away from the alveolar bone during drilling due to the lack of fixation.

Verhamme et al. (2015) [23] assessed the accuracy of implants with and without fixation screws. Interestingly, the author noted instances in which fixation pins were abandoned as they impeded surgeons during the procedure. While the use of fixation screws showed a statistical difference in buccolingual cervical deviations, the author argued that these screws do not necessarily enhance the stability of the surgical template, a viewpoint supported by other researchers such as Stübinger et al. (2014) [38]. Furthermore, Verhamme et al. (2015) [23] suggest that without fixation screws, surgeons have the advantage of continuous haptic feedback regarding the guide’s position.

Similarly, Kauffman et al. (2018) [39] compared the use of fixation pins with hand fixation, where they found a slight advantage in favor of the fixated group, although the difference was not substantial. It is worth noting that if the guide is not properly aligned from the start, the fixation tools may exacerbate the error [10]. According to Cassetta et al. (2014) [40] the accuracy during positioning of the surgical guide can be more affected by translational movement than by a rotational one.

The inclusion of physical stops, as discussed by some authors [24,27,28,29,30] serves to physically limit the depth of the osteotomy by restricting drill advancement only up to the level of the stopper. Arisan et al. (2010) [37] further assert that physical stops not only regulate the depth of the osteotomy but also constrain the direction of drilling.

Within Vercruyssen et al.’s. study (2014) [29], the Materialise^®^ Universal Group (Materialise NV, Leuven, Belgium) did not incorporate physical stops, whereas the Facilitate™ System (Dentsply Sirona, Charlotte, NC, USA) did utilize them. The study did not confirm a greater precision in the Facilitate™ System. However, it is important to note that depth deviations, which are purportedly mitigated by the use of stops, were not evaluated.

Another method that controls the direction and depth of the drill is through the use of guiding sleeves, which come in various systems. Some systems employ multiple surgical guides with sleeves of increasing diameters for each patient, while others utilize a single guide with removable sleeve inserts or integrated sleeves on the osteotomy drills [24,29].

Many researchers concur these discrepancies between the guide’s sleeve, drill guide, and drill can introduce deviations. These discrepancies, however, are needed to minimize the friction between instruments. In an in vitro study conducted by Horwitz et al. (2009) [41], it was discovered that the wear and tear of sleeves and drills over prolonged use contributes to increased deviations.

To address this challenge, Arisan et al. (2013) [30] propose that starting the osteotomy in parallel with the guide sleeve is crucial, as a non-parallel start has been shown to significantly increase deviations due to the rotational tolerance of the drills in the sleeves.

Moreover, in an in vitro study conducted by Koop et al. (2012) [42], it was noted that the employment of drill-hold sleeve inserts resulted in larger deviations for all measurements compared to handhold sleeve inserts. However, in Vercruyssen et al.’s investigation (2014) [29], where drill-hold sleeves were utilized, similar deviation values to those observed in Koop et al.’s (2012) [42] study were identified. This implies that the deviations observed in vitro may not be clinically significant.

According to Kholy et al. (2019) [43], reducing the drilling distance below the guided sleeve and increasing the length of the guided key above the sleeve can reduce both 3D and angular deviations. Nonetheless, longer drill sleeves require a larger mouth opening, which might not be suitable for all patients.

Sarhan et al. (2021) [26] conducted a comparison between cylindrical guiding holes and C-shaped ones. C-shaped guiding holes are said to facilitate a lateral drill placement, enhancing accessibility, particularly in posterior regions and in patients with restricted interarch distance. Moreover, C-shaped guiding holes aid in directing cooling irrigation to the osteotomy site during drilling. Despite the recommendation of a parallel osteotomy to reduce potential deviations, the design of the guiding holes, whether cylindrical or C-shaped, did not affect the accuracy of implant placement.

Additional factors beyond the control of clinicians may contribute to increased deviation values. For instance, a restricted mouth opening could result in the drill head being inclined more mesially or lingually during the procedure [26,30].

Vercruyssen et al. (2014) [29] noted in their study that in a patient with a limited mouth opening, the placement of the two most distal implants was not feasible. For this reason, several authors [31,32] have established inclusion criteria requiring a minimum mouth opening of at least 30 to 50 mm.

Smoking habits, known to cause thicker mucosa and reduced alveolar bone density, are also proposed to contribute to increased deviations during implant placement [30,44]. Variations in mucosal resilience between smokers and non-smokers might impact the degrees of freedom when placing a scanning prosthesis or a surgical guide [45].

However, Vercruyssen et al. (2014) [29] did not observe any influence of smoking on implant accuracy in their study, although the smoking population was relatively small.

In studies comparing the accuracy of implants placed in the maxilla versus the mandible [29,32], significantly greater deviations were observed in the mandible, particularly in terms of angular and coronal deviations. Cassetta et al. (2013) (2012) [35,44] suggest that this discrepancy is likely due to the combination of fixation screws and greater surface support in the maxilla, which minimize guide displacement during surgery. Safety margins should be higher in the mandible. Some studies [30,46] found no significant differences, while the Systematic Review by Marliere et al. [1] reported greater deviations in the maxilla. Future research should focus on clarifying the observed discrepancies between the maxilla and mandible, investigating the underlying causes of these variations.

Only one study [29] assessed the accuracy of bone-supported versus mucosa-supported surgical guides, finding no significant differences. Nevertheless, in a systematic review by Tahmaseb et al. (2018) [47], bone-supported guides exhibited significantly larger deviations compared to other types of guide support. These findings may shed light on why flapless approaches tend to have higher accuracy than flapped ones, as many procedures involving flap elevation are performed using bone-supported guides.

Vercruyssen et al. (2014) [29] also observed significantly lower deviations for anterior implants compared to posterior ones, a finding supported by other researchers [48,49].

When analyzing deviations in implant placement, four primary types are typically considered: coronal, apical, angular, and depth deviations.

Nomiyama et al. (2023) [28] reported the highest mean deviations for cervical and apical deviations, at 2.01 ± 0.77 mm and 2.41 ± 1.45 mm respectively, while Jaemsuwan et al. (2023) [31] observed the largest angular deviation at 4.98 ± 2.16°.

Cunha et al. (2021) [32] suggest that deviations less than 2 degrees may not be clinically significant in implant dentistry, though it is noteworthy that no studies achieved a mean angular deviation lower than 2 degrees. The relevance of angulation errors lies in their impact on prosthetic outcomes, as they have the potential to compromise the final prosthetic result. This holds particularly true for pre-made immediate dentures, as errors may prevent their proper alignment and fixation.

Arisan et al. (2013) [30] proposed that the implant shoulder’s location is restricted within the guidance tube, while the apical part of the implant has more freedom, possibly explaining why apical deviations tend to be higher than coronal deviations, a consistent finding across all selected studies. Additionally, it is suggested that apical deviation may increase with the implant tip’s added length. However, Vercruyssen et al. (2014) [29] did not find any influence of implant length on accuracy in their study.

Nomiyama et al. (2023) [28] proposed that the initial angulation may affect linear deviations, since the initial angulation has a direct correlation to apical deviations. Given that the apical area presents the highest risk of anatomical structure damage due to distant access by the dentist, it remains an important variable to be controlled.

Albiero et al. (2019) [27] conducted a comparison between a group of fresh extraction sockets and those already edentulous, noting a significant difference only in the apical deviation. In both groups, the apical deviation was notably influenced by coronal and angular deviations. This influence was markedly higher in the fresh extraction group, likely due to the lamina dura in this group directing the drill along the path of least resistance. Consequently, clinicians should exercise greater caution regarding drilling direction and angulation in fresh extraction sockets.

Depth deviations were also studied in some studies [23,24,26,27,28].

When it comes to depth values, their calculation can be ambiguous among studies. Sarhan et al. (2021) [26] and Verhamme et al. (2015) [23] depict positive values as indicating implants being positioned deeper than intended, while Albiero et al. (2019) [27] and Vercruyssen et al. (2016) [24] propose the opposite interpretation. The method employed by Nomiyama et al. (2023) [28] for determining depth deviations lacks clarity.

Verhamme et al. (2015) [23] observed that over half of the implants were positioned too superficially by at least 0.5 mm. This finding aligns with a previous study by Casseta et al. (2011) [50], which also reported a prevalence of implants placed too superficially. The results by Albiero et al. (2019) [27] and Vercruyssen et al. (2016) [24] also support this observation. Additionally, Nomiyama et al. (2023) [28] proposed that the lack of visibility of vital structures in the flapless approach might heighten the risk of depth deviation.

Two studies [23,24] analyzed MD and BL deviations. Verhamme et al. (2015) [23] suggest that breaking down 3D results into BL and MD directions provides more clinically relevant outcomes. Their findings indicate statistically significant differences observed in medial-lateral translation of the surgical guide at both the implant tip and shoulder in the BL direction and at the implant tip in the MD direction.

In the anterior-posterior direction, significant differences were found at the implant tip and shoulder only in the BL direction. Additionally, implant length demonstrated a statistical difference for angular deviation in the MD direction, with larger deviations observed at the implant tip.

Comparing these results with those of Vercruyssen et al. (2016) [24] may be challenging due to differences in the methodology used to measure MD and BL deviations. Nevertheless, measurements by Vercruyssen et al. (2016) [24] in the MD and BL directions align with findings from prior studies [51,52].

Regarding survival rates, Nomiyama et al. (2023) [28] reported a rate of 96.6%, while Arisan et al. (2013) [30] and Vercruyssen et al. (2016) [24] held rates of 98.1% and 98.8%, respectively. These findings align with other systematic reviews [53,54] that indicate rates ranging from 97.2% to 97.8%. Survival rates and the loss of implants were not reported in the other studies [23,26,27,29,31,32].

In comparing the precision of static fully guided surgery versus conventional implant surgery, studies have shown that guided surgery offers significantly greater accuracy than the freehand approach in fully edentulous patients [28,31].

A limited number of studies were analyzed to assess the impact of various factors on implant positioning accuracy, to ensure similarities in the conditions of the included studies. However, significant heterogeneity was noted among them, stemming from variations in study design, the methodology and software used for evaluating accuracy, the timing of outcome analysis, and clinical protocols. These discrepancies must be taken into consideration when interpreting the results of this systematic review and prevent the possibility of conducting a meta-analysis.

Future research should focus on the development and implementation of standardized methodologies and uniform conditions. By doing so, studies will yield more accurate and comparable results, which are essential to drawing reliable conclusions. Establishing consistent protocols will not only enhance the interpretability of findings but also contribute to the establishment of safety margins, ultimately improving patient outcomes and advancing the field.

## 5. Conclusions

This systematic review assessed the precision of static fully guided surgery in edentulous patients. It highlighted the importance of using the “backwards” technique for implant planning, considering bone and anatomical structures, and employing the flapless technique with guide fixation and a parallel osteotomy during surgery. Apical deviations exceed coronal ones, requiring careful attention to avoid damaging structures. Higher safety margins are needed in the mandible. While beneficial, static-guided surgery demands meticulous planning and execution, with further studies with similar methodologies needed to understand accuracy factors.

## Figures and Tables

**Table 1:** Clinical information obtained from the selected studies.

Authors	Year	Type of Study	Patients (n)	Mean Age	CAD-CAM Software	Implant Brand	Follow-Up
Sarhan et al. [26]	2021	Randomized Clinical Trial	12	NR	Blue Sky Bio^®^	Dentium^®^	Same day
Albiero et al. [27]	2019	Randomized Clinical Trial	20	58.9 (43–86)	SimPlant^®^	Dentsply^®^	1 week
Nomiyama et al. [28]	2023	Randomized Clinical Trial	29	62 (18–76)	Dental Slice Virtual Navigation^®^	Implacil^®^	10 days
Vercruyssen et al. [29]	2014	Randomized Clinical Trial	59	58 (NR)	SimPlant^®^	Astra Tech^®^	10 days
Verhamme et al. [23]	2015	Clinical Trial	25	59 (45–79)	Procera Clinical Design^®^	Nobel BioCare^®^	2 weeks
Jaemsuwan et al. [31]	2022	Clinical Trial	13	66 (51–75)	coDiagnostiX^®^	Straumann^®^	1 week
Cunha et al. [32]	2021	Clinical Trial	8	NR	P3Dental^®^	Easy Implant^®^	30 days
Arisan et al. [30]	2013	Randomized Clinical Trial	11	NR	SimPlant^®^	Thommen SPI Element^®^	2 months-mandible 3 months-maxilla
Vercruyssen et al. [24]	2016	Randomized Clinical Trial	15	60 (NR)	SimPlant^®^	Dentsply^®^	Same day

NR: Not referenced.

**Table 2:** Inclusion and exclusion criteria as well as type of deviations present in the selected studies.

Authors	Inclusion Criteria	Exclusion Criteria	Type of Deviation
Sarhan et al. [26]	Fully edentulous Mandibular ridge with sufficient height to accommodate 12 mm implants Buccolingual width of at least 6 mm	Narrow interarch distance Flabby soft tissues (more than 5 mm thickness)	3D Deviations
Albiero et al. [27]	Fully edentulous jaws or with compromised dentition (requiring extraction)	NR	3D Deviations
Nomiyama et al. [28]	Fully maxillary edentulous patients, with a minimum bone thickness of 5 mm Bone height of 9 mm for placing 6 implants	Pregnancy, lactation, antibiotic therapy within the past 6 months Medication that could alter osseointegration, such as anti-inflammatory drugs, bisphosphonates, or immunosuppressive drugs Need for grafting before or during surgery Prior regenerative procedures in the area designated for implant placement Patients with severe complications such as type 2 diabetes, cardiovascular and peripheral vascular diseases, neuropathies, and nephropathies	3D Deviations
Vercruyssen et al. [29]	Informed consent Minimum age of 18 years Extraction socket healing for at least 6 months Sufficient bone volume	Unlikely to be able to comply with study procedures History of intravenous bisphosphonate treatment Medical conditions that make implant placement unfavorable Current pregnancy Alcohol and/or drug abuse Major systemic diseases Untreated, uncontrolled caries and/or periodontal disease Prior exposure to local irradiation	3D Deviations
Verhamme et al. [23]	Extreme atrophy of the edentulous upper jaw Height and/or width of the alveolar ridge below 5 mm	NR	3D Deviations MD and BL Deviations
Jaemsuwan et al. [31]	Fully edentulous Need for at least two implants for restoration Sufficient bone volume for implant placement Mouth opening of at least 30 mm Suitable for all three protocols: (a) Freehand implant placement (b) Static CAIS (c) Dynamic CAIS Having a temporary denture for the planning phase	Systemic condition that might hinder osseointegration Clinical or radiographic pathology in the jaw bone Declination to sign a consent document	3D Deviations
Cunha et al. [32]	Fully edentulous jaws In good health, non-smoking, absence of degenerative and infectious diseases Classified as American Society of Anesthesiologists (ASA) I or II without systemic issues that would impede the surgical procedure Bone thickness of 5 mm in the buccolingual direction and a height of 10 mm in the alveolar ridge Mouth opening of 50 mm No need for bone regeneration procedures or bone grafts during the implant surgery	NR	3D Deviations
Arisan et al. [30]	Presence of >5 mm bone thickness A minimum of 5 mm attached mucosa	Poor systemic health condition, parafunctional behaviors, inadequate oral hygiene, insufficient alveolar bone volume, uncontrolled diabetes, recent head or neck irradiation, psychological disorders, or alcohol or tobacco or drug abuse	3D Deviations
Vercruyssen et al. [24]	Sufficient bone volume to place six implants in the edentulous maxilla Informed consent Age of 18 years or above Extraction sockets must be fully healed for a minimum of 4 months No previous bone augmentation procedures The mandibular dentition can vary, as long as an evenly distributed contact relationship with the new prosthesis in the maxilla can be achieved Accepting compliance with study procedures	Physical or psychological disorders that might hinder implant placement Heavy smoking (>10 cigarettes/day) Substance abuse including alcohol and/or drugs Physical disability that may impede oral hygiene practices	3D Deviations MD and BL Deviations

**Table 3:** Information regarding the guided surgical procedure and the accuracy of implant placement.

					Primary Deviations				Secondary Deviations	
Authors	Jaw	Implants * (*n*) (Analyzed) (*n*)	Flapless	Type of Surgical Guide	Cervical (mm)	Apical (mm)	Angular (°)	Depth (mm)	MD	BL
Sarhan et al. [26]	Mand	24 (24)	Yes	Mucosa-supported with fixation	Cyl: $\tilde{x}$ = 1.65 (0.91–4.54) Csh: $\tilde{x}$ = 1.27 (0.84–2.16)	Cyl: $\tilde{x}$ = 1.91 (0.77–6.68) Csh: $\tilde{x}$ = 2.46 (1.10–3.95)	Cyl: $\tilde{x}$ = 11.11 (4.22–14.76) Csh: $\tilde{x}$ = 11.04 (4.05–20.55)	Cyl: $\tilde{x}$ = 1.01 (0.28–2.54) Csh: $\tilde{x}$ = 1.69 (0.53–3.43)	NR	NR
Albiero et al. [27]	Both	114 (114)	Yes	Mucosa-supported with fixation	$\bar{x}$ = 1.20 ± 0.56 EA: $\bar{x}$ = 1.12 ± 0.52 FS: $\bar{x}$ = 1.28 ± 0.59	$\bar{x}$ = 1.51 ± 0.71 EA: $\bar{x}$ = 1.36 ± 0.68 FS: $\bar{x}$ = 1.65 ± 0.71	$\bar{x}$ = 3.30 ± 1.65 EA: $\bar{x}$ = 3.16 ± 1.79 FS: $\bar{x}$ =3.42 ± 1.52	$\bar{x}$ = 0.52 ± 0.85 EA: $\bar{x}$ = 0.51 ± 0.74 FS: $\bar{x}$ = 0.53 ± 0.94	NR	NR
Nomiyama et al. [28]	Max	87 (86)	Yes	Mucosa-supported with fixation	$\bar{x}$ = 2.01 ± 0.77	$\bar{x}$ = 2.41 ± 1.45	$\bar{x}$ =2.39 ± 0.79	$\bar{x}$ =1.67 ± 0.82	NR	NR
Vercruyssen et al. [29]	Both	212 (209)	No	Mucosa/Bone-supported with fixation	MatMu: $\bar{x}$ = 1.23 ± 0.06 * MatBo: $\bar{x}$ = 1.60 ± 0.92 FacMu: $\bar{x}$ = 1.38 ± 0.64 * FacBo: $\bar{x}$ = 1.33 ± 0.82	MatMu: $\bar{x}$ = 1.57 ± 0.71 MatBo: $\bar{x}$ = 1.65 ± 0.82 FacMu: $\bar{x}$ = 1.60 ± 0.70 * FacBo: $\bar{x}$ = 1.50 ± 0.72	MatMu: $\bar{x}$ = 2.86 ± 1.60 MatBo: $\bar{x}$ = 3.79 ± 2.36 FacMu: $\bar{x}$ = 2.71 ± 1.36 * FacBo: $\bar{x}$ = 3.20 ± 2.70	NR	NR	NR
Verhamme et al. [23]	Max	150 (150)	Yes	Mucosa-supported with and without fixation	$\bar{x}$ = 1.963 ± 0.232	$\bar{x}$ = 2.288 ± 0.269	$\bar{x}$ = 3.926 ± 0.414	$\bar{x}$ = −0.584 ± 0.155	Cervical: $\bar{x}$ = 1.270 ± 0425 Apical: $\bar{x}$ = 1.494 ± 0.466 Angular: $\bar{x}$ = 2.504 ± 0.573 Depth: −$\bar{x}$ = 0.602 ± 0.315	Cervical: $\bar{x}$ = 0.757 ± 0.180 Apical: $\bar{x}$ = 0.987 ± 0.279 Angular: $\bar{x}$ = 2.484 ± 0.568 Depth: −$\bar{x}$ = 0.571 ± 0.291
Jaemsuwan et al. [31]	Both	20 (20)	No	Mucosa-supported with fixation	$\bar{x}$ = 1.40 ± 0.72	$\bar{x}$ = 1.66 ± 0.61	$\bar{x}$ = 4.98 ± 2.16	NR	NR	NR
Cunha et al. [32]	Both	60 (60)	Yes	Mucosa-supported with fixation	$\bar{x}$ = 0.68 ± 0.36 Mand: $\bar{x}$ = 0.79 ± 0.42 Max: $\bar{x}$ = 0.61 ± 0.30	$\bar{x}$ = 0.82 ± 0.39 Mand: $\bar{x}$ = 0.92 ± 0.39 Max: $\bar{x}$ = 0.75 ± 0.37	$\bar{x}$ = 2.04 ± 1.21 Mand: $\bar{x}$ = 2.44 ± 1.43 Max: $\bar{x}$ = 1.77 ± 0.0.95	NR	NR	NR
Arisan et al. [30]	Both	108 (102)	Yes	Mucosa-supported with fixation	CBCT: $\bar{x}$ =0.81 ± 0.32 MSCT: $\bar{x}$ = 0.75 ± 0.32	CBCT: $\bar{x}$ = 0.87 ± 0.32 MSCT: $\bar{x}$ = 0.80 ± 0.35	CBCT: $\bar{x}$ = 3.47 ± 1.144 MSCT: $\bar{x}$ = 3.30 ± 1.08	NR	NR	NR
Vercruyssen et al. [24]	Max	90 (90)	Yes	Mucosa-supported with fixation	$\bar{x}$ = 0.9 (0.1–4.5)	$\bar{x}$ = 1.2 (0.2–4.9)	$\bar{x}$ = 2.7 (0.6–6)	$\tilde{x}$ = 0.11 (−3.18–1.79)	$\tilde{x}$ = −0.12 (−2.25–1.33)	$\tilde{x}$ = −0.2 (−2.17–1.65)

Note: The number of implants considered for each study only accounts for placed implants where a static fully guided surgical guide was utilized. Legend: With Fixation: Stabilization of the surgical guide with fixation/osteosynthesis pins or screws; Cyl: Cylindrical guiding hole; Csh: C-shaped guiding hole; EA: Edentulous arch; FS: Fresh extraction socket; MatMu: Materialise universal^®^/mucosa; MatBo: Materialise universal^®^/bone; FacMu: Facilitate™/mucosa; FacBo: Facilitate™/bone; CBCT: Cone beam computed tomography; MSCT: Multislice spiral computed tomography; Mand: Mandible; Max: Maxilla; NR: Not referenced

## Data Availability

The data that support the findings of this study are available on request from the corresponding author.

## Supplementary Materials

The following supporting information can be downloaded at: https://www.mdpi.com/article/10.3390/jcm13175178/s1, File S1: Articles that were excluded and the reason for exclusion.
