# Supplementary material for: Accuracy of Implant Guided Surgery in Fully Edentulous Patients: Prediction vs. Actual Outcome—Systematic Review
Source: J Clin Med. 2024 Aug 31;13(17):5178. doi: 10.3390/jcm13175178 (PMC11396269; doi:10.3390/jcm13175178)
Supplement: Supplementary file 1 [file jcm-13-05178-s001.zip › jcm-3162801-supplementary.pdf]

Supplementary table - Articles that were excluded and the reason for exclusion

ARTICLE EXCLUDED	REASON FOR EXCLUSION
DIGITAL SMILE DESIGNED COMPUTER-AIDED SURGERY VERSUS TRADITIONAL WORKFLOW IN "ALL ON FOUR" REHABILITATIONS: A RANDOMIZED CLINICAL TRIAL WITH 4-YEARS-FOLLOW-UP (CATTONI ET AL., 2021)	Doesn't evaluate implant deviations
COMPUTER-GUIDED VS FREEHAND PLACEMENT OF IMMEDIATELY LOADED DENTAL IMPLANTS: 5-YEAR POSTLOADING RESULTS OF A RANDOMISED CONTROLLED TRIAL (TALLARICO M FAU - ESPOSITO, ESPOSITO M FAU - XHANARI, XHANARI E FAU - CANEVA, CANEVA M FAU - MELONI, & MELONI, 2018)	Doesn't evaluate implant deviations
AN RCT COMPARING PATIENT-CENTRED OUTCOME VARIABLES OF GUIDED SURGERY (BONE OR MUCOSA SUPPORTED) WITH CONVENTIONAL IMPLANT PLACEMENT (VERCRUYSSEN, DE LAAT A FAU - COUCKE, COUCKE W FAU - QUIRYNEN, & QUIRYNEN, 2014)	Doesn't evaluate implant deviations
IMPLANT- AND PATIENT-CENTRED OUTCOMES OF GUIDED SURGERY, A 1-YEAR FOLLOW-UP: AN RCT COMPARING GUIDED SURGERY WITH CONVENTIONAL IMPLANT PLACEMENT (VERCRUYSSEN, VAN DE WIELE G FAU - TEUGHELS, ET AL., 2014)	Doesn't evaluate implant deviations
REHABILITATION OF FULL-MOUTH EDENTULISM: IMMEDIATE LOADING OF IMPLANTS INSERTED WITH COMPUTER-GUIDED FLAPLESS SURGERY VERSUS CONVENTIONAL DENTURES: A 5-YEAR MULTICENTER RETROSPECTIVE ANALYSIS AND OHIP QUESTIONNAIRE (MARRA, ACOCELLA A FAU - ALESSANDRA, ALESSANDRA R FAU - GANZ, GANZ SD FAU - BLASI, & BLASI, 2017)	Doesn't evaluate implant deviations
FIVE-YEAR RESULTS OF A RANDOMIZED CONTROLLED TRIAL COMPARING PATIENTS REHABILITATED WITH IMMEDIATELY LOADED MAXILLARY CROSS-ARCH FIXED DENTAL PROSTHESIS SUPPORTED BY FOUR OR SIX IMPLANTS PLACED USING GUIDED SURGERY (TALLARICO, MELONI, CANULLO, CANEVA, & POLIZZI, 2016)	Doesn't evaluate implant deviations
ACCURACY OF POSITIONING OF IMPLANTS INSERTED USING A MUCOSA-SUPPORTED STEREOLITHOGRAPHIC SURGICAL GUIDE IN THE	Observational study (wrong type of study)

EDENTULOUS MAXILLA AND MANDIBLE (CASSETTA M FAU - GIANSANTI, GIANSANTI M FAU - DI MAMBRO, DI MAMBRO A FAU - STEFANELLI, & STEFANELLI, 2014)	
COMPUTER-GUIDED VERSUS FREE-HAND PLACEMENT OF IMMEDIATELY LOADED DENTAL IMPLANTS: 1-YEAR POST- LOADING RESULTS OF A MULTICENTRE RANDOMISED CONTROLLED TRIAL (POZZI A FAU - TALLARICO, TALLARICO M FAU - MARCHETTI, MARCHETTI M FAU - SCARFÒ, SCARFÒ B FAU - ESPOSITO, & ESPOSITO, 2014)	Doesn't distinguish/evaluate fully edentulous patients
IS IT POSSIBLE TO IMPROVE THE ACCURACY OF IMPLANTS INSERTED WITH A STEREOLITHOGRAPHIC SURGICAL GUIDE BY REDUCING THE TOLERANCE BETWEEN MECHANICAL COMPONENTS? (CASSETTA, DI MAMBRO A FAU - GIANSANTI, GIANSANTI M FAU - STEFANELLI, STEFANELLI LV FAU - BARBATO, & BARBATO, 2013)	Only evaluates angle deviations
HOW DOES AN ERROR IN POSITIONING THE TEMPLATE AFFECT THE ACCURACY OF IMPLANTS INSERTED USING A SINGLE FIXED MUCOSA-SUPPORTED STEREOLITHOGRAPHIC SURGICAL GUIDE? (CASSETTA, DI MAMBRO, GIANSANTI, STEFANELLI, & BARBATO, 2014)	Observational study (wrong type of study)
THE INFLUENCE OF THE TOLERANCE BETWEEN MECHANICAL COMPONENTS ON THE ACCURACY OF IMPLANTS INSERTED WITH A STEREOLITHOGRAPHIC SURGICAL GUIDE: A RETROSPECTIVE CLINICAL STUDY (CASSETTA, DI MAMBRO, DI GIORGIO, STEFANELLI, & BARBATO, 2015)	Only evaluates angle deviations
ACCURACY OF IMPLANT POSITION WHEN PLACED USING STATIC COMPUTER-ASSISTED IMPLANT SURGICAL GUIDES MANUFACTURED WITH TWO DIFFERENT OPTICAL SCANNING TECHNIQUES: A RANDOMIZED CLINICAL TRIAL (KIATKROEKKRAI, TAKOLPUCKDEE, SUBBALEKHA, MATTHEOS, & PIMKHAOKHAM, 2020)	Doesn't distinguish/evaluate fully edentulous patients
PRECISION OF SLEEVELESS 3D DRILL GUIDES FOR INSERTION OF ONE-PIECE CERAMIC IMPLANTS: A PROSPECTIVE CLINICAL TRIAL (SCHNUTENHAUS S FAU - VON KOENIGSMARCK ET AL., 2018)	Study not made in humans
COMPARISON OF THE ACCURACY OF IMPLANT POSITION FOR TWO-IMPLANTS SUPPORTED FIXED DENTAL PROSTHESIS USING STATIC AND DYNAMIC COMPUTER-ASSISTED IMPLANT SURGERY: A	Doesn't distinguish/evaluate fully edentulous patients

RANDOMIZED CONTROLLED CLINICAL TRIAL (YIMARJ ET AL., 2020)	
ACCURACY OF FREEHAND VERSUS GUIDED IMMEDIATE IMPLANT PLACEMENT: A RANDOMIZED CONTROLLED TRIAL (CHANDRAN, GOYAL, MITTAL, & GEORGE, 2023)	Doesn't distinguish/evaluate fully edentulous patients
DIGITAL IMPLANT PLACEMENT ACCURACY: A CLINICAL STUDY ON A FULLY-GUIDED FLAPLESS SINGLE-UNIT IMMEDIATE-LOADING PROTOCOL (PIROOZ, ATRI, GHOLAMI, & BAYAT, 2023)	Doesn't distinguish/evaluate fully edentulous patients
THE ACCURACY OF IMPLANT PLACEMENT USING OPENED VERSUS CLOSED SLEEVE COMPUTER SURGICAL GUIDE A SPLIT MOUTH TECHNIQUE (ABDALLAH, KADDAH, & ELKHADEM, 2021)	Doesn't distinguish/evaluate fully edentulous patients
ACCURACY OF IMPLANT SURGICAL GUIDES FABRICATED USING COMPUTER NUMERICAL CONTROL MILLING FOR EDENTULOUS JAWS: A PILOT CLINICAL TRIAL (CHAI ET AL., 2020)	Cohort study (wrong type of study)
ACCURACY OF EDENTULOUS COMPUTER-AIDED IMPLANT SURGERY AS COMPARED TO VIRTUAL PLANNING: A RETROSPECTIVE MULTICENTER STUDY (VINCI ET AL., 2020)	Observational study (wrong type of study)
IMPACT OF SURGICAL TEMPLATE ON THE ACCURACY OF IMPLANT PLACEMENT (XU, YOU, ZHANG, LIU, & PENG, 2016)	Doesn't distinguish/evaluate fully edentulous patients
ACCURACY OF A DEDICATED BONE-SUPPORTED SURGICAL TEMPLATE FOR DENTAL IMPLANT PLACEMENT WITH DIRECT VISUAL CONTROL (SUN, LUEBBERS, AGBAJE, KONG, ET AL., 2015)	Static fully guided surgery guides aren't used
ACCURACY OF DENTAL IMPLANT PLACEMENT USING CBCT-DERIVED MUCOSA-SUPPORTED STEREOLITHOGRAPHIC TEMPLATE (SUN, LUEBBERS, AGBAJE, SCHEPERS, ET AL., 2015)	Case Series (wrong type of study)
ACCURACY OF IMAGE-FUSION STEREOLITHOGRAPHIC GUIDES: MAPPING CT DATA WITH THREE-DIMENSIONAL OPTICAL SURFACE SCANNING (WIDMANN ET AL., 2015)	Study not made in humans
A CLINICALLY RELEVANT ACCURACY STUDY OF COMPUTER-PLANNED IMPLANT PLACEMENT IN THE EDENTULOUS MAXILLA USING MUCOSA-SUPPORTED SURGICAL TEMPLATES (VERHAMME, MEIJER GJ FAU - BOUMANS, ET AL., 2015)	Case Series (wrong type of study)

ACCURACY OF COMPUTER-AIDED TEMPLATE-GUIDED ORAL IMPLANT PLACEMENT: A PROSPECTIVE CLINICAL STUDY (BERETTA, POLI, & MAIORANA, 2014)	Doesn't distinguish/evaluate fully edentulous patients
A CLINICALLY RELEVANT VALIDATION METHOD FOR IMPLANT PLACEMENT AFTER VIRTUAL PLANNING (VERHAMME ET AL., 2013)	Case Series (wrong type of study)
THREE-DIMENSIONAL ACCURACY OF GUIDED IMPLANT PLACEMENT: INDIRECT ASSESSMENT OF CLINICAL OUTCOMES (PLATZER, BERTHA, HESCHL, WEGSCHEIDER, & LORENZONI, 2013)	Doesn't distinguish/evaluate fully edentulous patients
THE INTRINSIC ERROR OF A STEREOLITHOGRAPHIC SURGICAL TEMPLATE IN IMPLANT GUIDED SURGERY (CASSETTA, DI MAMBRO, GIANSANTI, STEFANELLI, & CAVALLINI, 2013)	Doesn't distinguish/evaluate fully edentulous patients
ACCURACY IN STATIC GUIDED IMPLANT SURGERY: RESULTS FROM A MULTICENTER RETROSPECTIVE CLINICAL STUDY ON 21 PATIENTS TREATED IN THREE PRIVATE PRACTICES (LUONGO ET AL., 2024)	Doesn't distinguish/evaluate fully edentulous patients
